# Mechanical mapping of mammalian follicle development using Brillouin microscopy

**DOI:** 10.1038/s42003-021-02662-5

**Published:** 2021-09-27

**Authors:** Chii Jou Chan, Carlo Bevilacqua, Robert Prevedel

**Affiliations:** 1 Cell Biology and Biophysics Unit, European Molecular Biology Laboratory, Heidelberg, Germany; 2 Collaboration for joint PhD degree between EMBL and Heidelberg University, Faculty of Biosciences, Heidelberg University, Heidelberg, Germany; 3 Developmental Biology Unit, European Molecular Biology Laboratory, Heidelberg, Germany; 4 Epigenetics and Neurobiology Unit, European Molecular Biology Laboratory, Monterotondo, Italy; 5 Molecular Medicine Partnership Unit (MMPU), European Molecular Biology Laboratory, Heidelberg, Germany; 6 Present Address: Mechanobiology Institute, National University of Singapore, Singapore, Singapore; 7 Present Address: Department of Biological Sciences, National University of Singapore, Singapore, Singapore

**Keywords:** Oogenesis, Biophysical methods, Imaging, Biophysics

## Abstract

In early mammalian development, the maturation of follicles containing the immature oocytes is an important biological process as the functional oocytes provide the bulk genetic and cytoplasmic materials for successful reproduction. Despite recent work demonstrating the regulatory role of mechanical stress in oocyte growth, quantitative studies of ovarian mechanical properties remain lacking both in vivo and ex vivo. In this work, we quantify the material properties of ooplasm, follicles and connective tissues in intact mouse ovaries at distinct stages of follicle development using Brillouin microscopy, a non-invasive tool to probe mechanics in three-dimensional (3D) tissues. We find that the ovarian cortex and its interior stroma have distinct material properties associated with extracellular matrix deposition, and that intra-follicular mechanical compartments emerge during follicle maturation. Our work provides an alternative approach to study the role of mechanics in follicle morphogenesis and might pave the way for future understanding of mechanotransduction in reproductive biology, with potential implications for infertility diagnosis and treatment.

## Abstract (web summary)

Chan et al. apply Brillouin microscopy to study changes in tissue material properties during mammalian follicle development. Focusing on the Brillouin loss tangent which is independent of the refractive index and mass density, they find that mammalian folliculogenesis is characterised by region-specific changes in tissue micro-viscosity driven by cell differentiation and extracellular matrix remodelling.

## Introduction

**Fig. 1:**
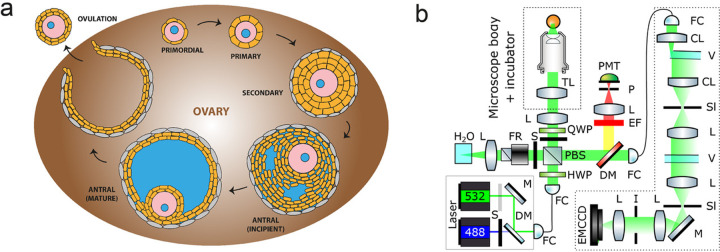
Characterizing mechanical properties of ovaries ex vivo with Brillouin microscopy. **a** Schematic of the developmental cycle of follicles during mouse folliculogenesis. A follicle consists of the oocyte (pink) with its nucleus (blue), surrounded by the somatic cells (orange) and theca cells (gray). The oocyte grows in size during transition to secondary follicle stage, followed by the emergence of fluid-filled lumen (blue) in the antral follicle stage. The oocyte is eventually released during ovulation, and upon fertilization undergoes embryo development. The interstitial tissues of the ovaries comprise of stromal cells, extracellular matrix, and vasculature. **b** Schematic of the confocal Brillouin imaging setup. L = lens, TL = tube lens, PMT = photomultiplier, P = pinhole, EF = emission filter, DM = dichroic mirror, FC = fiber coupler/collimator, QWP = quarter waveplate, HWP = half waveplate, PBS = polarizing beam splitter, S = shutter, FR = Faraday rotator, CL = cylindrical lens, V = VIPA, Sl = adjustable slit, V = VIPA, I = iris.

In mammalian development, folliculogenesis describes the progression of a number of small primordial follicles into large preovulatory follicles with functional oocytes in the ovaries (Fig. 1a). Upon activation, the primordial follicle transitions into primary state, when the surrounding somatic cells (granulosa cells (GCs)) become cuboidal and undergo extensive proliferation. It then develops into a secondary follicle with multiple layers of GCs, basal lamina, and a theca layer. This is followed by the formation of fluid-filled antral follicle, and ovulation where the oocyte is released from the ovary. While hormonal signaling is known to impact antral follicle formation onwards, early stages of preantral follicle development are known to rely on intra-follicular signaling^1^. Recently there is emerging evidence that mechanical stress imposed by the extracellular matrix (ECM) plays a role in the oocyte development, such as the activation of primordial follicles^2,3^. Furthermore, it has long been hypothesized that the ovarian cortex and the inner medulla have distinct stiffness^4^, although this has not been formally tested. We reason that this is because appropriate tools for quantifying tissue material properties in vivo do not yet exist or face important limitations. Currently, Atomic Force Microscopy (AFM)^5^ and micropipette aspiration^6,7^ are most often used to infer mechanical properties, such as elasticity or viscosity in the micrometer regime at cellular surfaces or in superficial tissues. A recent AFM study reported regional differences in ovarian stiffness^8^, although the work was carried out in bisected ovaries which may release tissue stress and perturb the mechanical properties of the ovaries^9^. Other approaches such as optical coherence elastography and stress sensors involving liquid droplets and deformable gel beads have also been developed to infer tissue mechanical properties and stress in 3D^10–14^. However, all these methods are invasive which could perturb the sample, rely on mechanical models to extract local elastic parameters, and are unable to provide a high-resolution spatial map of the measured mechanics.

Here, Brillouin microscopy offers an optical and thus non-invasive approach to infer mechanical properties of cells and tissues at high spatial resolution in 3D. It relies on the interaction and inelastic scattering of monochromatic laser light from thermally driven acoustic phonons at high frequencies^15–17^. The scattered light spectrum is indicative of the material’s sound velocity and thus local mechanical properties. In particular, it gives access to the (micro-)elasticity and viscosity through the measurement of the longitudinal modulus and acoustic wave attenuation at the micrometer-scale. Brillouin microscopy is an emerging technique that has been successfully applied to questions in cell and tissue biology^18–20^, development^21–24^ as well as within a medical context^25–27^. These studies have typically compared tissue stiffness based on the shift of the Brillouin spectrum alone (see Supplementary Note 1), although more sophisticated analysis approaches have also been explored to distinguish individual tissue components from the spectrum^20,28^. While reporting the shift alone has become common and widespread practice in the field, the Brillouin shift alone only informs about elastic properties and is also dependent on the material’s refractive index and mass density^16^. Therefore, in situations where the cells or tissues are visco-elastic, or the refractive index and mass density are unknown, it is impossible to separate the contributions of mechanical properties and the refractive index or density to any observed spatial or temporal changes in the Brillouin frequency shifts. To address this intrinsic limitation, in this study we focus on a less ambiguous metric, the Brillouin loss tangent (BLT), which allows us to single out the mechanical contribution to the observed Brillouin signals.

In this study, we examined the mechanical properties of mouse ovaries in situ using confocal Brillouin microscopy, focusing on the interstitial tissues and follicles during development. We showed that mammalian folliculogenesis is accompanied by a spatio-temporal change in intra-follicular and interstitial mechanics, largely dominated by micro-viscosity changes during cell differentiation and extracellular matrix remodeling.

## Results

### Quantifying cell and tissue material properties with Brillouin loss tangent (BLT)

To address the above-mentioned technical limitation with respect to characterizing mechanical properties in 3D, we utilized Brillouin microscopy, which is based on high-resolution spectroscopic measurements of the light scattered by the material under investigation (see Fig. 1b, Methods and Supplementary Note 1). In particular, the backscattered light spectrum, i.e., its peak position and linewidth, provides information about the longitudinal modulus at hypersonic (GHz) frequencies. From the spectrum’s frequency shift, *ν*, and its full-width at half-maximum (FWHM), Γ, the storage and loss moduli of the sample can be inferred as *M*′ = *ρ*(*νλ*/2*n*)^2^, and *M*′′ = *ρ*Γ*ν(λ*/2*n*)^2^, which accounts for the elastic and viscous behavior of the sample^16^. Here, *ρ* is the density and *n* the material refractive index within the scattering volume, and *λ* the incident wavelength. As is evident from their definition, both the local refractive index and density are central to a proper quantification of the mechanical properties. However, techniques such as tomographic phase microscopy^29,30^ or optical diffraction tomography^31–33^ that can provide information about optical properties in three dimensions and in situ, do not work in thick and highly scattering tissues such as the ovary.

In order to decouple the effect of mechanical and optical properties in Brillouin microscopy, here we utilize the so-called Brillouin loss tangent (BLT), defined as tan(φ) = *M*′′/*M*′ = Γ/*ν*. By its definition, the BLT does not depend on the sample refractive index and density and thus provides a simple approach to determine whether mechanical properties are the main contributor to observed changes in the Brillouin spectrum and their spatial maps. Such spatial mapping of BLT was first carried out on human epithelial tissue^20^, albeit on cryosectioned tissues that may not reflect the true mechanical state of living tissues^34^. Similar approach has also been used to study bulk mechanical properties in tumor spheroids^26^ and cellulose fibers^35^. From a mechanical viewpoint, the loss tangent can be understood as the relative contribution of effective viscosity to longitudinal modulus within the probed, micrometer-scale region of the sample. It is therefore a measure of how well energy is stored or dissipated in the tissue. In general, a high loss tangent indicates a more pronounced energy dissipation of the (local) phonons, indicative of higher micro-viscosity^36^. On the contrary, a small loss tangent indicates a higher structural order in the material, which incurs less acoustic attenuation and reflects higher ‘elasticity’ (see Supplementary Note 1 for further discussion).

To validate the loss tangent as a meaningful parameter in comparing mechanical properties of biological samples, we imaged and compared the Brillouin frequency shift, linewidth, and BLT of the nucleoli and nucleoplasm in mouse embryos at the preimplantation stage. As shown in Supplementary Fig. 1, the nucleoli have significantly higher BLT compared to the nucleoplasm, suggesting that the nucleoli contained more dissipative elements compared to the nucleoplasm at the microscopic scale. This is consistent with the emerging evidence that the nucleoli are membraneless organelles that exhibit liquid-like properties such as fusion and phase separation in diverse biological contexts^37–39^. Similar reports of nucleoli being more viscous than the nucleoplasm was also reported in the germ cells of *C. elegans*, as shown by stimulated Brillouin scattering microscopy^24^.

### The ovary exhibits regional difference in tissue mechanical properties

**Fig. 2:**
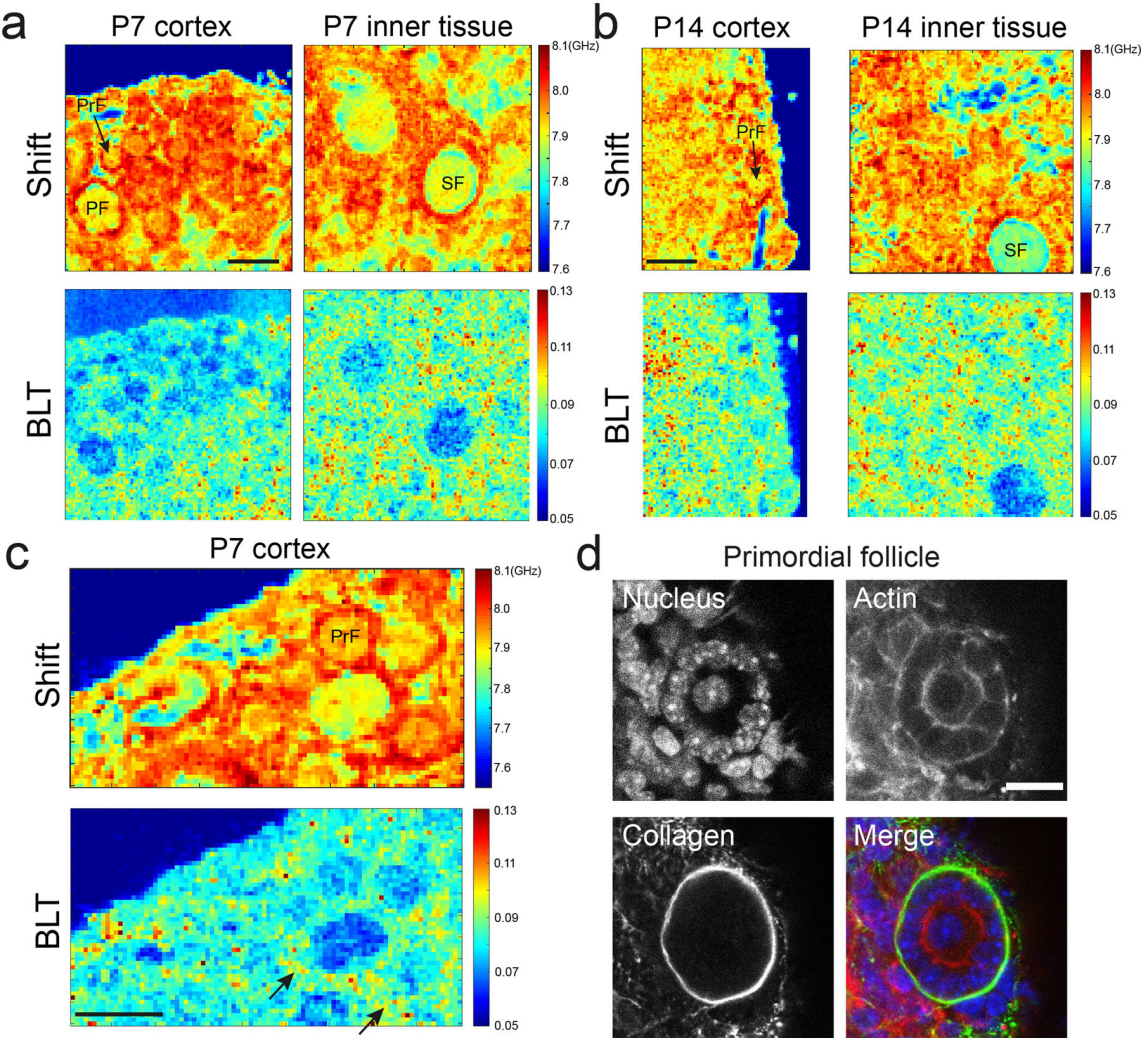
Ovary tissue is characterized by regional differences in mechanical properties. **a** Maps of Brillouin shift and the corresponding BLT for a P7 ovarian cortex and the inner tissue. Both images were obtained from the same ovary. Primordial follicles (PrF) and primary follicles (PF) reside primarily in the cortex while the secondary follicles (SF) tend to be found in the inner part of the ovary, which shows a higher interstitial BLT. **b** Maps of Brillouin shift and BLT for a P14 ovarian cortex and the inner tissue. Similar to P7 ovaries, higher PLT is associated with the inner tissue of the ovaries, compared to the cortex. **c** Zoom in view of P7 ovarian cortex showing higher BLT of cells (black arrows) surrounding the oocyte, which may be associated with the presence of collagen surrounding the primordial follicle, as shown in **d**. Scale bar = 40 μm in **a**, **b**, and **c**, and 15 μm in **d**.

We first investigated if there exists any regional difference in the ovarian mechanical properties during folliculogenesis (Fig. 2a, b). While the data showed significant variations of Brillouin shift across the tissues, which likely correspond to the different structural components, shift alone is not sufficient to inform mechanical variations due to its dependence on optical properties, as discussed above. We therefore further analyzed the linewidth and BLT. Interestingly, we found that the regional difference in BLT arises mainly from a change in the linewidth (Supplementary Fig. 2), suggesting that the ovarian mechanics is largely dominated by changes in micro-viscosity. To exclude this coming from experimental uncertainties and artefacts, we further determined our linewidth measurement precision (96 MHz, see Supplementary Fig. 3), which is about 1/10 of a typical linewidth encountered in the ovarian tissue and smaller than any temporal changes observed. Other spurious effects such as the high NA geometry employed in our microscope, or improper lineshape fitting, were also found to not explain the relatively large linewidth heterogeneity observed in our data (see Methods and Supplementary Fig. 4).

Focusing on the BLT (Fig. 2a, b), we found a visibly higher BLT in the P7 and P14 ovarian interior compared to the cortex where the primordial follicles typically reside. This suggests that the ovarian cortex may be more elastic than the ovarian interior, which is populated with the larger secondary follicles and stroma that consist of collagen fibers, fibroblasts, and vasculature^40^. Zooming into the primordial follicles, we observed that they are characterized by oocytes with a low BLT, surrounded by a somatic cell layer with higher BLT (Fig. 2c). Notably, we also observed a ring of significantly higher BLT, possibly associated with the presence of a basement membrane that encapsulates the primordial follicles^1^ (Fig. 2d).

### Mechanical compartments emerge during follicle maturation

**Fig. 3:**
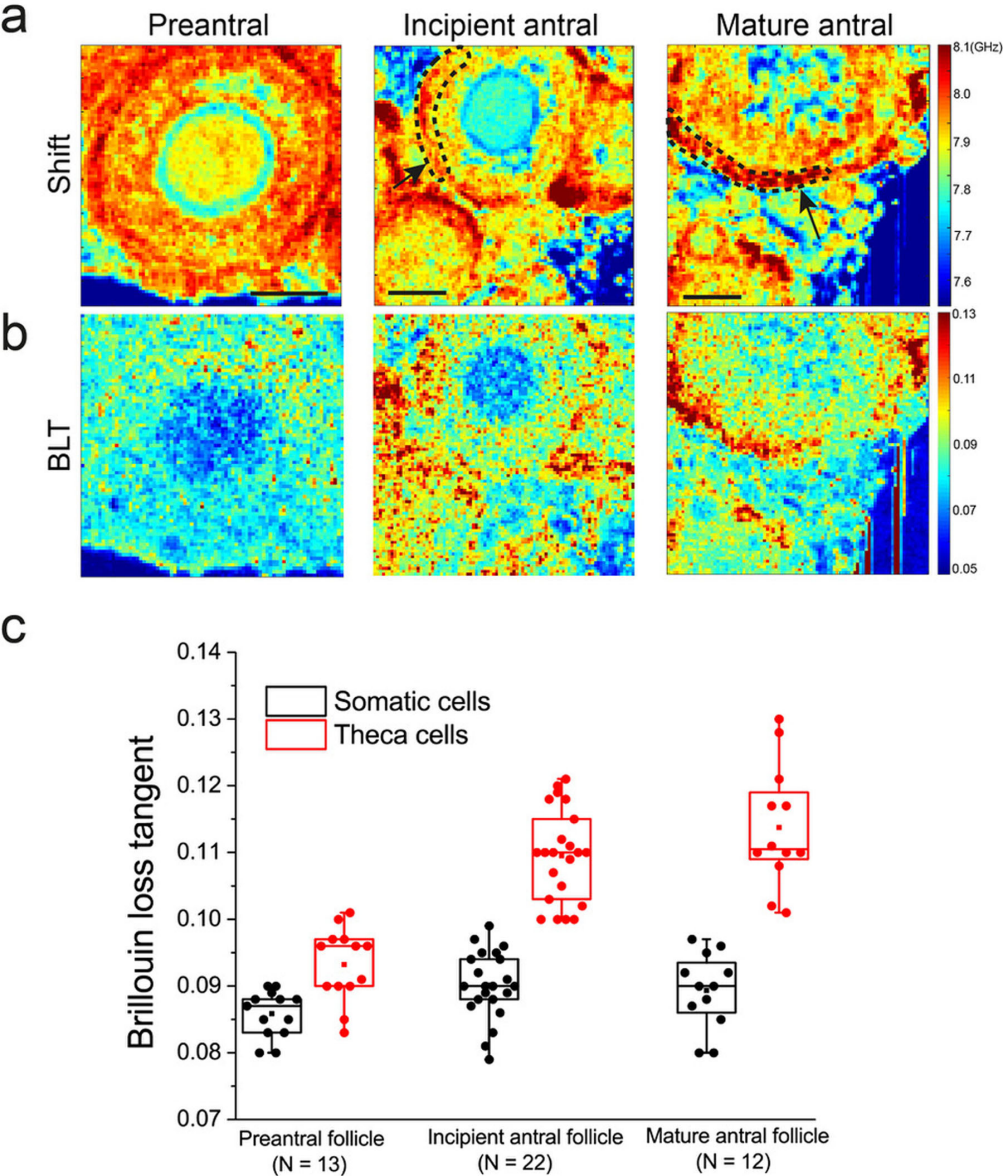
Distinct mechanical compartments emerge during follicle maturation. **a** Row shows representative maps of Brillouin shift for follicles at distinct stages of folliculogenesis. Scale bar = 40 μm. **b** Corresponding maps of BLT for **a**. **c** Boxplot of BLT for the outer theca cells versus the inner somatic cell layer of follicles at various stages of development. Each data point corresponds to the average signal of somatic or theca cells in one follicle. Black arrows indicate the follicles under consideration, black dashed lines indicate the region of interest for the theca cell layer.

We next performed mechanical mapping of follicles from the secondary to the antral follicles stage, which is marked by the presence of large fluid-filled lumen. Here, remarkably, we found that during this transition an outer tissue ‘shell’ with a significantly higher BLT starts to form and encapsulate the follicle (Fig. 3b, c). This mechanical compartmentalization becomes most pronounced at the mature follicle stage. The typical width of this ‘shell’ is larger than 10 µm, suggesting that they originate from cellular entities, possibly from the theca cells. This is also consistent with the fact that the theca cells become more established at the antral follicle stage, compared to the secondary follicle stage^41^.

**Fig. 4:**
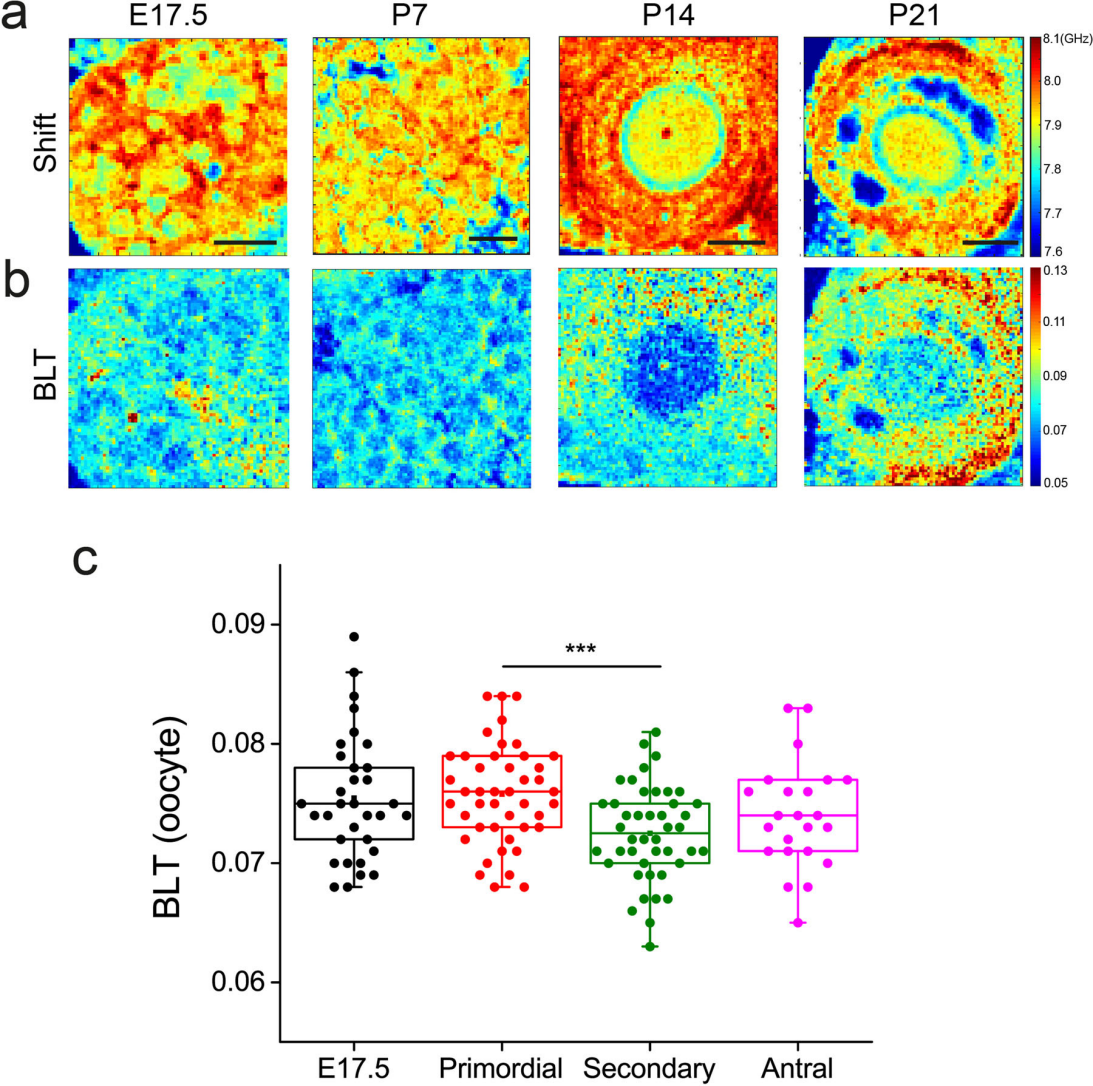
Ooplasm exhibits more liquid-like behavior at the early stages of oogenesis. **a** Row shows representative maps of Brillouin shift for oocytes from fetal ovaries (E17.5), primordial follicles (P7), secondary follicle (P14) and antral follicle (P21) ovaries. Scale bar = 40 μm. Blue regions within the antral follicle (P21) indicate fluid lumena. **b** Corresponding Brillouin loss tangent for **a**. **c** Boxplot of BLT for oocytes at various stages of follicle development. Each data point corresponds to the average signal of one oocyte. *N* = 35, 45, 45, 21 for E17.5 germ cells and oocytes from primordial, secondary, and antral follicle stage, respectively. ***P < 0.001.

Of note, Brillouin imaging allows us to visualize the microlumina or singly resolved lumen in live antral follicles (see blue regions within the follicles in the shift maps Figs. 3a and 4a), which is not readily detected by dyes or other common microscopy methods. This ability to image luminal structures in living tissues in a label-free manner based on the different tissue/fluid material properties is a unique strength of Brillouin microscopy. Future longitudinal studies of cultured antral follicles with Brillouin microscopy may reveal key mechanisms of lumen initiation and coalescence during antral follicle morphogenesis.

Following early studies showing changes in the ultrastructure of the oocytes at various stages of growth^42,43^, we next quantify the ooplasmic micro-viscoelasticity during folliculogenesis (Fig. 4). Interestingly, we observed that the primordial follicles in all stages of follicle development exhibit a small but significantly higher BLT compared to the larger oocytes from the secondary and antral follicles (Fig. 4c). Germ cells from the fetal ovaries (E17.5), which are not surrounded by the somatic cells, also have a relatively high ooplasmic BLT compared to the secondary or antral follicles. These results suggest that the early oocytes contain more mechanical heterogeneities in the ooplasm compared to the mid- and late stage oocytes.

### Higher BLT correlates with interstitial ECM deposition

**Fig. 5:**
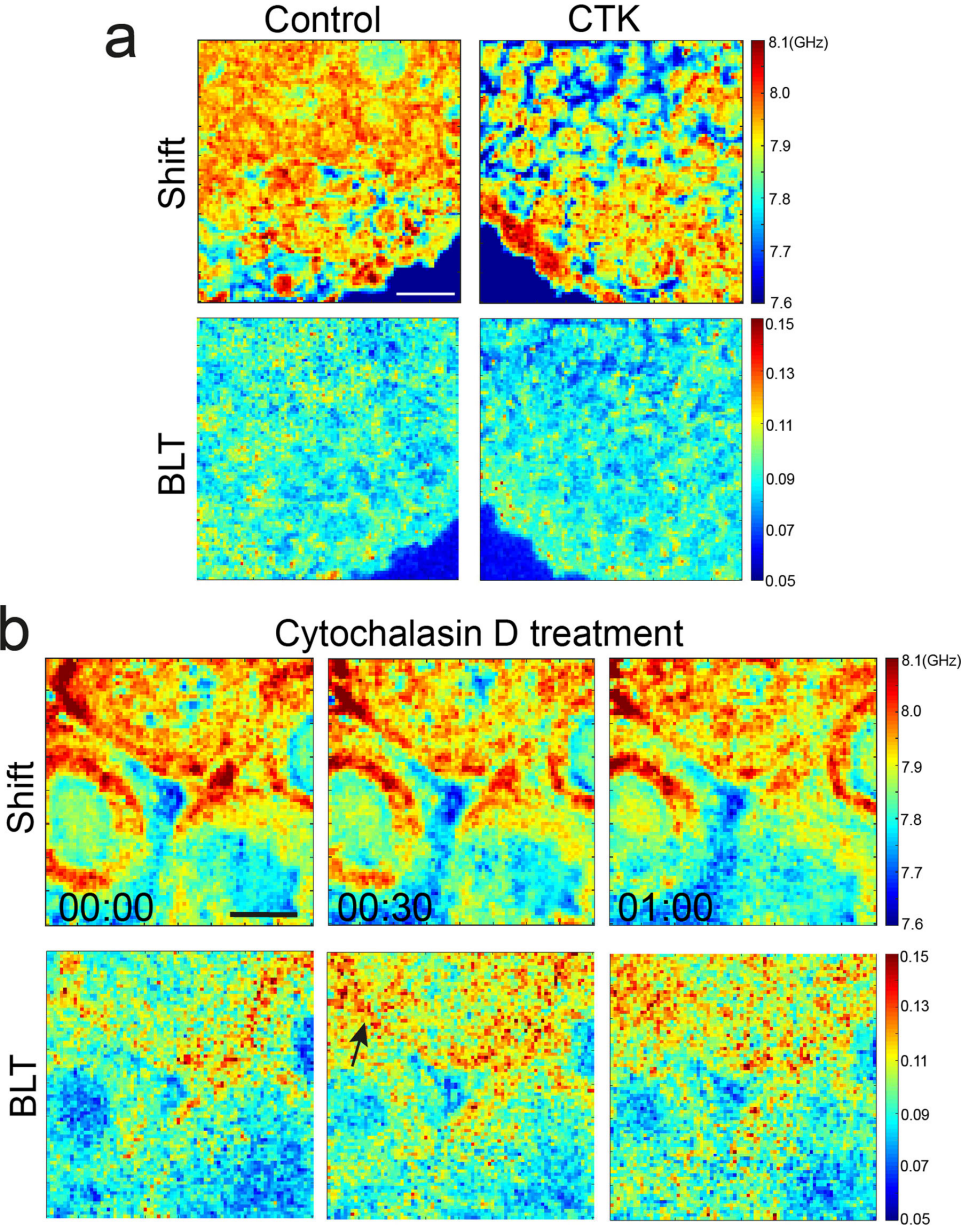
Disruption of extracellular matrix and actin cytoskeleton impacts interstitial viscoelasticity. **a** Top row: Brillouin shift of a P14 ovary when treated with CTK, which digests collagen matrix. CTK leads to a clear increase in the interstitial space (blue) between the primordial follicles at the cortex. Bottom row: Corresponding BLT maps. Scale bar = 40 μm. **b** Top row: Brillouin shift of a P14 ovary when treated with cytochalasin D, a pharmacological inhibitor that disrupts actin cytoskeleton. Lower row: Corresponding BLT maps showing that a disruption of actin cytoskeleton leads to higher BLT (black arrow) and decreased elasticity of the interstitial tissues. Scale bar = 40 μm. Time denotes hh:mm.

To further investigate the origin of changes in BLT signals, we studied the distribution of fibrillar collagen, a known contributor to tissue viscoelasticity^44^, in mouse ovaries at various stages of follicle development. Using Second Harmonic Generation (SHG) imaging, we observed clear interstitial collagen signals in P14 and P21 ovaries but not in P7 ovaries (Supplementary Fig. 5). Primordial, secondary, and antral follicles are embedded in the collagen network, which became denser and more bundle-liked in the P21 ovaries. This progressive increase in interstitial collagen network during follicle maturation correlates well with the increase in interstitial BLT (Fig. 2a, b), suggesting that ECM deposition may contribute to the ovarian mechanical heterogeneities. Indeed, when we treated the ovaries with CTK, a solution containing collagenase that digest the ECM (see Methods), we observed clear disruption of tissue integrity shown by the increased interstitial spacing and disconnected follicles (Fig. 5a).

We further performed immunofluorescence staining, which showed that the actin cytoskeleton exists abundantly within the follicles and the surrounding stroma (Fig. 2d). To ascertain the role of actin cytoskeleton in ovarian tissue viscoelasticity, we treated the ovaries with cytochalasin D, an inhibitor of actin polymerization. This led to a partial increase in intra and inter-follicle tissue BLT (Fig. 5b), indicating that the ovarian tissue is becoming more liquid-like, consistent with the role of actin cytoskeleton in maintaining cellular elasticity^18^.

## Discussion

In this study, to the best of our knowledge, we demonstrated the first spatial and temporal mechanical mapping of mammalian ovarian development in situ, with micron-level spatial resolution. From a biological perspective, our work represents a step towards addressing the mechanical aspects of folliculogenesis, which has gained recognition due to emerging evidence that mechanical stress in the ovary can influence the quality of the growing oocytes through ECM stiffness^45,46^, Hippo signaling pathway^47,48^ and increased fibrosis^49,50^. A recent related work by Remer et al.^24^ also reported mechanical mapping of *Caenorhabditis elegans* nematodes at the reproductive stage, but focused more on the technical novelty of stimulated Brillouin scattering microscopy rather than the biomechanical changes that occur during development.

It is important to note that Brillouin scattering probes the material properties in the GHz frequency range, which is in contrast to many existing methods currently utilized in biomechanics. This means that the elasticity (longitudinal modulus) measured by Brillouin scattering will typically assume much higher values (in the GPa range) for common biomaterials, compared with the widely used Young’s modulus *E* (often in the kPa range). In contrast to the conventionally used Brillouin shift, which is convoluted with the influence of refractive index and mass density, the BLT is independent of these parameters and therefore serves as an unambiguous metric to compare regional differences in tissue viscoelasticity across space and time. While a recent study has combined Brillouin microscopy with optical diffraction tomography to uniquely determine the refractive index and thus quantitative longitudinal modulus of cells on 2D substrates^31,33^, it remains challenging to extend this approach to thick (>100 µm) tissues. In such scenarios, utilizing and quantifying the BLT may be the best alternative to investigate the mechanical properties in a non-invasive and label-free fashion.

Importantly, as a relatively new measure, the interpretation of BLT warrants some discussion. The BLT has been shown to be inversely proportional to the acoustic attenuation length^36^. A high BLT therefore indicates stronger acoustic attenuation and energy dissipation and implies a higher micro-viscosity of the material (also see Supplementary Note 1). Hence the nucleoli having a higher BLT than the nucleoplasm (Supplementary Fig. 1) suggests that the former contains more elastic heterogeneities. Meanwhile, the *zona pellucida* (Supplementary Fig. 1) and the ooplasm (Fig. 4) having much lower BLT implies that these structures contain less elastic heterogeneities or mechanical disorder that contribute to a less ‘viscous’ microenvironment.

In our work we found a strong correlation between the BLT and the width (e.g., Supplementary Figs. 2 and 6b). This can be explained by the presence of *heterogeneous linewidth broadening*, which occurs when the scattering volume (~1.5 µm in the largest dimension in our case) is larger than the size of the elastic heterogeneities (for example, organelles or matrix pore, also see Supplementary Note). In this case the final spectrum is the sum of Brillouin peaks originating from the different sub-regions, and the overall spectral width is accordingly increased. In this case, the shift is the weighted average of these peaks, thus the two parameters are not correlated, which is also evident from our data (Supplementary Fig. 6a). Furthermore, this is likely not a spurious or technical artefact due to our reduced (VIPA-based) spectrometer resolution, as related work by Caponi et al. also found a strong contribution of the linewidth to the BLT metric with a higher-resolution spectrometer^51^.

We have shown that the connective tissues of the ovarian cortex are more elastic compared to the interior of the ovary, which appears more viscous-like (Fig. 2) and is enriched with fibrillar collagen (Supplementary Fig. 5). Indeed, while there is substantial research to support the elastic role of ECM, the porous nature of the ECM in causing substantial viscous dissipation has only been recognized more recently^44^. Furthermore, the higher BLT associated with the ECM is largely attributed to the variation in the linewidth (Supplementary Fig. 2) and the microscopic viscosity. Interestingly, our finding is consistent with several reports showing that hydrophobic hydration around the collagen can lead to higher BLT and phonon attenuation^17,20,52^ (see Supplementary Note 1 for further discussion). Here, multimodal approaches incorporating infrared or Raman spectroscopy which can specifically map bulk water as well as hydrated molecular species, could be useful for further investigations, as demonstrated by other groups^20,53,54^. From the biological perspective, given that the dormant primordial follicles are known to reside primarily in the cortex while the larger secondary follicles are found in the ovarian interior, this raises the intriguing possibility that the regional difference in ECM viscoelasticity may influence the follicle positioning and tissue patterning in vivo.

At the intra-follicular level, we showed that the periphery of the primordial follicles has a higher BLT associated with the basement membrane (Fig. 2c). In view of recent evidence that the basement membrane plays a role in maintaining primordial follicle dormancy^1^, our finding suggests that this may involve some mechanotransduction pathways triggered by the distinct basement membrane viscoelasticity. In secondary and antral follicles, the theca cells were found to exhibit higher micro-viscosity than the somatic cells (Fig. 3). This may be due to the fact that the theca cells are associated with increased expression of hyaluronic acid, which is known to promote tissue hydration^50^. The theca cell layer may provide a soft microenvironment and serve as a mechanical ‘sponge’ to buffer the oocyte and somatic cells against external mechanical stress. The exact origin and functions for the theca cell mechanics will be exciting topics for future studies. The changes in the ooplasmic BLT as the follicles mature to the late follicle stage (Fig. 4) suggest a change in the cytoplasmic ultrastructure and its micro-viscoelasticity. Interestingly, oocytes are known to undergo dramatic volumetric expansion (~10-fold) during secondary follicle development. Whether this rapid growth in oocyte size leads to ooplasmic dilution and changes in the ooplasmic viscoelasticity remains to be investigated in the future, possibly with the use of other techniques such as microrheology^55^. Future ultrastructural studies of the mechanical heterogeneities within the follicles and interstitial ECM, such as the use of electron microscopy will help to further understand the origin of these mechanical heterogeneities.

Overall, our work provides a proof-of-concept use of Brillouin microscopy in mapping out mammalian follicle’s mechanical environment in living ovaries. We foresee such non-invasive and 3D imaging technique harbors great potential for future investigation of dysregulated mechano-signaling pathways in reproductive ageing^24^ and ovarian diseases (e.g., polycystic ovary syndrome)^56^.

## Methods

### Ovary work and sample mounting

Embryonic and postnatal mice ovaries were obtained in the animal facility at the European Molecular Biology Laboratory, with permission from the institutional veterinarian overseeing the operation (ARC number 2020-01-06RP). The animal facilities are operated according to international animal welfare rules (Federation for Laboratory Animal Science Associations guidelines and recommendations). All ovaries were imaged within 3 h post dissection. Ovaries were imaged at less than 80 μm depth to avoid low SNR arising from tissue scattering. The illuminating laser power was 5 mW which was optimized to achieve optimal signals while maintaining sample viability. Ovary viability under 532 nm laser illumination was confirmed by repeated Brillouin imaging on the same slice which yielded identical maps of Brillouin shift and BLT within the ovaries.

### Imaging setup

The setup of the confocal Brillouin microscope has been described previously^57^, and its schematic is depicted in Fig. 1b. To image the ovaries in physiological conditions, the microscope body (Zeiss Axiovert 200 M) is equipped with a custom-built incubation chamber that can actively control the temperature, CO_2_ and O_2_ levels, and monitor humidity. The sample is positioned on a 3-axes Piezo translational stage (P-545.3R8H, Physik Instrumente) that allows point-scanning with sub-micrometer precision. The laser source for Brillouin imaging is a 532-nm CW single mode laser (Torus, Laser Quantum). The setup includes also a 488-nm diode laser (Omicron Luxx 488-60) used as an excitation source of GFP-like fluorophores. The two lasers are combined, by means of a dichroic mirror, and coupled into the same PM single-mode fiber to ensure collinearity and a clean beam profile at the output. After collimation the light passes through a half waveplate (HWP) followed by a polarizing beam splitter (PBS): the rotation of the HWP determines the ratio of intensities of the beams transmitted and reflected by the PBS, that are delivered respectively to the sample and to a cuvette filled with water for calibration. Two shutters, controlled via a self-written LabVIEW software, allow to collect a calibration spectrum every 50 points. The periodic calibration helps correct for the drift of the laser wavelength over time. The laser is focused on the sample by a 1.0 NA oil objective that gives a spatial resolution of 0.30 μm × 0.30 μm × 1.76 μm (FWHM), as measured from the edge response of the Brillouin amplitude at an oil water interface^58^. The backscattered light is collected by the same objective and, after being reflected by the PBS, is coupled into a single mode fiber and delivered to the Brillouin spectrometer. A narrowband bandpass filter (Semrock LL01-532) before the fiber coupler reflects the emitted fluorescence while transmitting the Brillouin signal. The fluorescence signal is detected by a photomultiplier tube (Thorlabs PMT1001) preceded by a pinhole that ensures confocality.

The Brillouin spectrometer is based on a two-stage virtually imaged phased array configuration^57^, featuring two VIPAs with a nominal free spectral range of 30 GHz and spectral resolution of 520 MHz. The addition of a Lyot stop^59^ before the camera (iXon DU897, EMCCD camera; Andor Technology) brings the suppression of elastically scattered light to ~65 dB. To quantify the precision of the linewidth measurements we utilized a tissue phantom since repeatedly measuring the width over a particular tissue region can be confounded by tissue movement and photodamage. For the tissue phantom we chose an intralipid solution (SIGMA I141-100Ml) in water which can mimic the same scattering and attenuation properties of tissues^60^. In brief, we estimated the tissue attenuation length in the ovary by fitting an exponential to the intensity of the Brillouin signal measured at four different depths within an ovary. Doing so yielded an attenuation length of ~70 μm at our 532 nm wavelength (Supplementary Fig. 3a). Using different concentrations of intralipid solution and a similar exponential fit approach over four different z-slices, we found that 16.7% v/v intralipid solution mimics the same attenuation length as that of the tissue. We then performed a linewidth measurement with the same laser power (5 mW) and depth (70 μm) as employed in the ovary tissue imaging experiments. We measured the spectral linewidth of the intralipid solution 400 times and quantified the uncertainty by the FWHM of the so-obtained histogram (Supplementary Fig. 3b). The uncertainty of the width was found to be 96 MHz, which is about 1/10 of a typical linewidth measured in an ovarian tissue. The same analysis in the same conditions was performed on the shift and the precision was found to be 32 MHz.

Since spectral resolution of the spectrometer is comparable to the typical linewidth of biological matter, deconvolution of the spectrum is advisable to obtain a sensible value for the true linewidth. Deconvolution is equivalent to a subtraction of the instrument response from the measured linewidth in case both spectra can be described by Lorentzian functions. To ascertain that a Lorentzian distribution best represents the experimental data, we have computed the Akaike information Criterion (AIC), a metric that is widely used for statistical analysis^61^, to compare the appropriateness of the different models (Lorentzian, Voigt and NA-Corrected^62^) in fitting our experimental Brillouin spectra. The larger the deviation of the model AIC from the Lorentzian AIC, the poorer is the fit of the model with respect to the Lorentzian model. Our analysis (Supplementary Fig. 4) showed that the Lorentzian model is a better fit compared to the Voigt’s model, while the NA-Corrected model is an equally good fit compared to the Lorentzian model. We therefore chose the Lorentzian fit as it is computationally less expensive and allowed us to perform simple deconvolution by subtracting the measured spectral resolution (as determined from the FWHM of the Rayleigh scattered laser) from the linewidth fitted to the experimental spectra.

We note that other experimental effects can further contribute to linewidth broadening: As discussed in the work by Mattana et al.^53^, the use of high NA optics causes a slight shift and broadening of the Brillouin peak due to the dependence of the Brillouin shift on the scattering vector $q=2{nk}\,{{\sin }}[(\pi -\alpha )/2]$ where ${\alpha }_{{MAX}}={{\arcsin }}({NA}/n).$ The increase in linewidth can be approximated by $\varDelta \nu /\nu =({q}_{{MAX}}-{q}_{{MIN}})/q=1-{{\sin }}[(\pi -\alpha )/2]\approx 5 \%$ for our NA. That corresponds to 20 MHz for water. Experimentally we have found that the difference in linewidth between 0.85NA and 0.34NA in our microscope is ~45 MHz. We therefore chose not to correct for broadening and spectral shifts due to high NA optics since the effect is comparatively small. Furthermore, such corrections would be exact only for isotropic samples and the broadening is affecting all the experimental points in the same way. Therefore such subtle corrections would not alter the general findings and conclusions of this work.

### Data analysis

The acquired scattering spectra were analyzed in real time with a custom-written Labview program by fitting Lorentzian functions to the spectral data. This yielded the position, width, and intensity of the Brillouin peaks. Pixel size was chosen to be 2 μm, an optimal compromise balancing resolution and Field-of-view (FOV) which was chosen to be 180 × 180 µm unless otherwise stated. Overall image acquisition time is typically around 15 min. Fitting may be noisy due to low SNR in deep tissues, hence only images or ROIs were selected for subsequent analysis whose datapoints displayed a fit with an *R*^2^ greater than 0.92. The images of Brillouin shift and linewidth were smoothened with Gaussian filter (radius 0.5 pixels, ImageJ), and the BLT data were generated using MATLAB. ROI for extra-follicular tissues, oocytes, somatic cells, and theca cells, as well the nucleoli and nucleoplasm in mouse embryos, were selected by checking the co-localization of the BLT and Brillouin shift, which showed a better contrast for these cellular structures.

### Statistics and reproducibility

All statistical analysis was performed using the software Origin (version 8.5.6; OriginLab, Northampton, MA). Significances for data displaying normal distributions were calculated with unpaired Student’s *t* test (two-tailed, unequal variance). All box plots extend from the 25^th^ to 75^th^ percentiles (horizontal box), with a line at the median and whiskers extending to max/min data points. No statistical methods were used to define sample size, which is chosen arbitrarily. Each experiment was repeated at least three times using different batches of ovaries on different days. Authors were blinded during experiments (mice were chosen randomly), but not blinded during analysis because images where the signal to noise ratio for the Brillouin spectra were too low were excluded. After selection, the parameters for different experiments were measured at random.

### Second harmonic generation imaging and analysis

Ovaries from P7, P14 mice, and mice older than P21 were harvested and placed in a glass slide with conical well, filled with 200 μl of PBS and covered with #1.5 coverslip (Thermo Fisher). SHG imaging of mouse ovaries was performed on Zeiss NLO LSM780 microscope equipped with a fs-pulsed laser. Ovaries were excited with a wavelength of 800 nm through a 25X (0.8 NA, water) objective. SHG signals from the tissues were captured in the backward direction in the autofluorescence channels of the microscope.

### Immunofluorescence staining

Ovaries were fixed with 4% PFA at 4 °C for 1 h. Following washing with PBS (1 h), samples were placed in blocking solution (PBS + 0.1% BSA (Sigma, 9647) + 0.3% Triton X-100 (Sigma, T8787)) and kept. Samples are then placed in blocking solution added with primary antibody and kept at 4 °C overnight. The samples were then washed three times with PBST (PBS + 0.1% Tween 20) for 1 h each time, before being kept at 4 °C overnight in secondary antibody diluted in blocking solution. Phalloidin and DAPI diluted 1:1000 in Blocking One Histo were added at this stage. Ovaries were then washed three times in PBST for 1 h each time, before being mounted in Fluoro-KEEPER antifade reagent (Nacalai) followed by imaging.

Primary antibody against collagen IV (Millipore, AB756P) was used at 1:100. Secondary antibodies targeting rabbit immunoglobulin-coupled Alexa Fluor 546 (Invitrogen, A10040) was used at 1:200. Alexa Fluor 633-coupled phalloidin (Invitrogen, R415) was used at 1:50. DAPI (Invitrogen, D3751) was used at 1:1000.

### Pharmacological inhibition

Following a similar protocol as a recent study^2^, CTK reagent was prepared as 1 mM CaCl_2_, collagenase type IV (0.1 mg/ml), 20% KSR (Invitrogen), and 0.025% trypsin EDTA (Invitrogen). Ovaries were treated with PBS or CTK for 1 h at 37 °C in a CO_2_ incubator before imaging. Cytochalasin D (Sigma) was used at a final concentration of 20 μM.

### Reporting summary

Further information on research design is available in the Nature Research Reporting Summary linked to this article.

## Supplementary information

Supplementary Information

Description of Additional Supplementary Files

Supplementary Data 1

Supplementary Data 2

Reporting Summary

## Data Availability

The datasets generated and analyzed in the current study are available from the corresponding authors upon reasonable request. Raw data for Figs. 3c and 4c are presented in Supplementary Data 1 and 2, respectively.

## Code availability

The code used for generating all the data in the current study are availabe from the corresponding authors upon reasonable request.
