# Supplementary material for: Mechanical mapping of mammalian follicle development using Brillouin microscopy
Source: Commun Biol. 2021 Sep 27;4:1133. doi: 10.1038/s42003-021-02662-5 (PMC8476509; doi:10.1038/s42003-021-02662-5)
Supplement: Supplementary file 1 — Supplementary Information [file 42003_2021_2662_MOESM1_ESM.pdf]

Supplementary Information

Supplementary Note 1: Mechanical interpretation of the Brillouin spectrum

Here we provide a brief discussion on the Brillouin spectrum. We discuss the interpretation of the Brillouin shift, linewidth and the loss tangent in terms of phonon propagation and dissipation. For further information, we refer to recent reviews[16,17,36].

Brillouin shift

For acoustic phonons (travelling density waves) having frequencies higher than the internal relaxation dynamics of the molecules, the probed region behaves effectively as a 'stiff' material. The Brillouin shift $\nu = Vq$, where V is the acoustic sound velocity and q is the wavevector in the scattering volume (that depends on the refractive index). V is also related to the mass density and the longitudinal elastic modulus M' through $V = \sqrt{M'/\rho}$, where ρ is the mass density. Hence the spectral shift directly informs the material elastic properties at GHz frequencies, provided the mass density and refractive index are known.

Brillouin line width

The line width Γ of the Brillouin peak contains information about the different phonon attenuation processes in the materials, which are classified as dynamic or static processes. For dynamic processes, the phonon energy is exchanged (via relaxation process) with the internal degrees of freedom of the materials. This process is particularly relevant in homogeneous viscoelastic samples, where the origin of this *homogeneous broadening* is associated with intrinsic viscous response of the sample. Indeed, the apparent longitudinal viscosity η is related to the line width of the Brillouin spectrum, Γ, through $\eta = \rho\Gamma/q^2$. The viscosity is defined 'apparent' as it is not static and depends on the frequency of the Brillouin peak. In the case of the static process, the phonon propagation can also be hindered by the presence of elastic heterogeneities (for example, organelles, matrix pores, etc.) in the materials. When the measurement length scale (typically set by the point spread function) is larger than the length scale of these elastic heterogeneities, a distribution of Brillouin peaks can be present, each characterized by a given elastic constant. In this case the measured spectrum is the sum of Brillouin peaks from the different sub-regions, which can contribute to significant *heterogeneous broadening* of the measured linewidth, in case the different components are not spectrally resolvable.

Brillouin loss tangent (BLT)

The functional meaning of BLT, despite its use in some previous studies[20,26,35], has not been formally discussed in detail. There are two approaches to interpret the BLT. One is based on its mathematical equivalence to the ratio of M'' (longitudinal loss modulus) and M' (longitudinal

elastic modulus). Here, a higher BLT indicates that at the probed frequency, the intrinsically viscoelastic tissue exhibits more fluid-like behavior compared to its solid-like property. The second way to understand the BLT is based on its direct inverse proportionality to the phonon mean free path, as described in Mattarelli *et al*[36]. Hence a higher BLT can be interpreted as a smaller phonon propagation length and stronger acoustic attenuation, which can be affected by dynamic dissipative processes (internal relaxation processes enhanced by tissue hydration) or elastic heterogeneities (e.g. amorphous or biological materials). BLT is therefore related to the *microstructural* composition of the material. For example, in the oocytes of the present study, the ooplasm and *zona pellucida* have significantly lower BLT compared to the somatic cells and interstitial ECM. This implies that the acoustic attenuation is weaker here, possibly due to a more 'ordered' microstructure in these materials. Interestingly, work by Palombo *et al*[20] reported a higher BLT associated with damping of acoustic phonons in bound water in collagen I and collagen-rich connective tissues. An alternative interpretation for the higher BLT observed in the interstitial ECM and the theca cells may therefore be attributed to the bulk water retained in these structures.

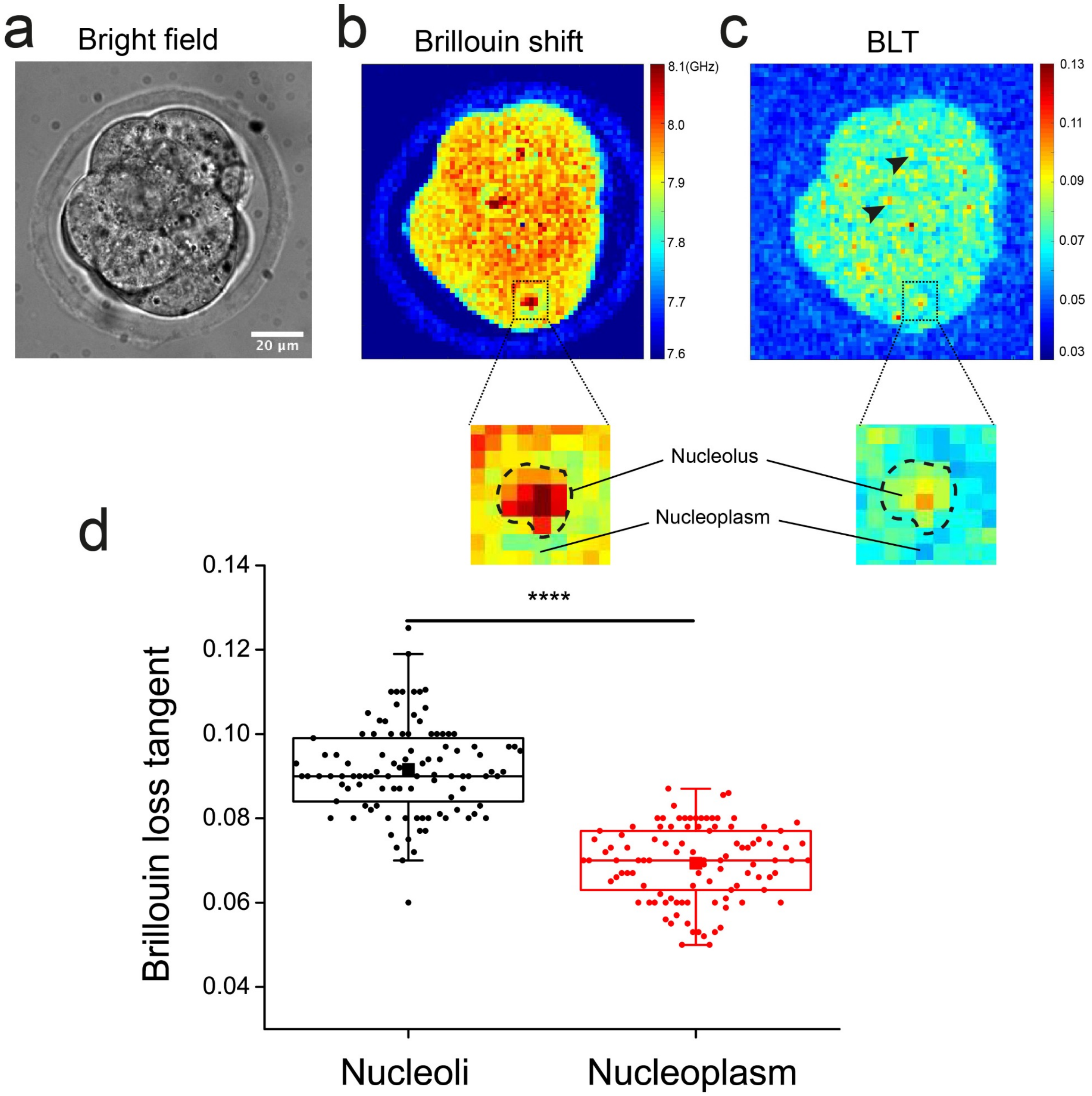

Supplementary Figure 1: Cell nucleoli have distinctly higher relative viscous components than that of the nucleoplasm. a. Brightfield image of an 8-cell stage mouse embryo. **b.** Map of Brillouin shift for the same mouse embryo in **a**, showing clearly the nuclei and nucleoli in three of the blastomeres. **c.** Corresponding Brillouin loss tangent for (a). Black arrowheads indicate the nucleoli while the boxes indicate zoom out regions of nucleoli (dashed black lines) and nucleoplasm. The regions for nucleoli and nucleoplasm were determined from the Brillouin shift data (b), which shows a better contrast for these structures. **d.** Brillouin loss tangent for nucleoli compared to the nucleoplasm. Data pooled from 102 mouse embryos at various stages of preimplantation. ****$P < 0.0001$. Same observation of the nucleoli having a higher BLT than the nucleoplasm was also reported for the secondary follicles in the mouse ovary (Fig. 4b, P14), and in *C. elegans* nematodes[24].

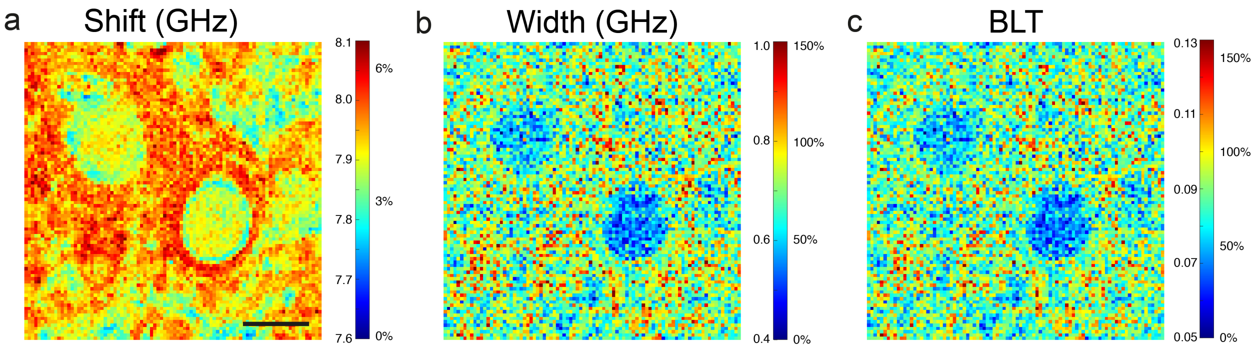

Supplementary Figure 2: Changes in BLT are mainly due to a change in the Brillouin linewidth and tissue micro-viscosity. Brillouin shift (**a**), width (**b**) and BLT (**c**) for a P7 ovarian interior (**Fig. 1a** in the main texts). Scale bar = 40 µm. Heat map bars indicate absolute values of the shift and width (left side of the bar) and percentage change relative to the medium (7.6 GHz and 0.4 GHz, right side of the bar). The relative change in BLT is approximately the sum of the relative changes of the shift and width. We therefore infer that the main contribution to changes in BLT is the linewidth and hence the effective micro-viscosity of the tissue.

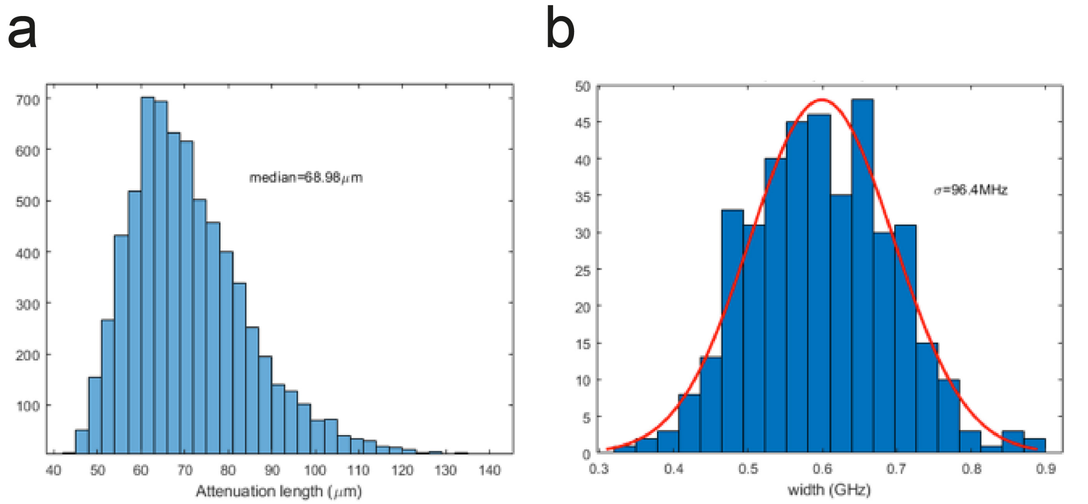

Supplementary Figure 3: Measurement of the attenuation length and linewidth precision.
a. The laser attenuation length in the ovary (P15) is calculated by fitting an exponential to the intensity of the Brillouin signal, measured on four different z-planes of an ovarian tissue. The median attenuation length was found as 69.0 µm Data is pooled from all the pixels in an image..
b. Histogram of linewidth measurement using a 16.7% v/v intralipid solution which mimics the same attenuation length as in ovarian tissue (a) (see Methods). Histogram contains 400 independent measurements. The uncertainty of the width is found to be 96 MHz, which is about 1/10 of a typical linewidth found in the ovarian tissue.

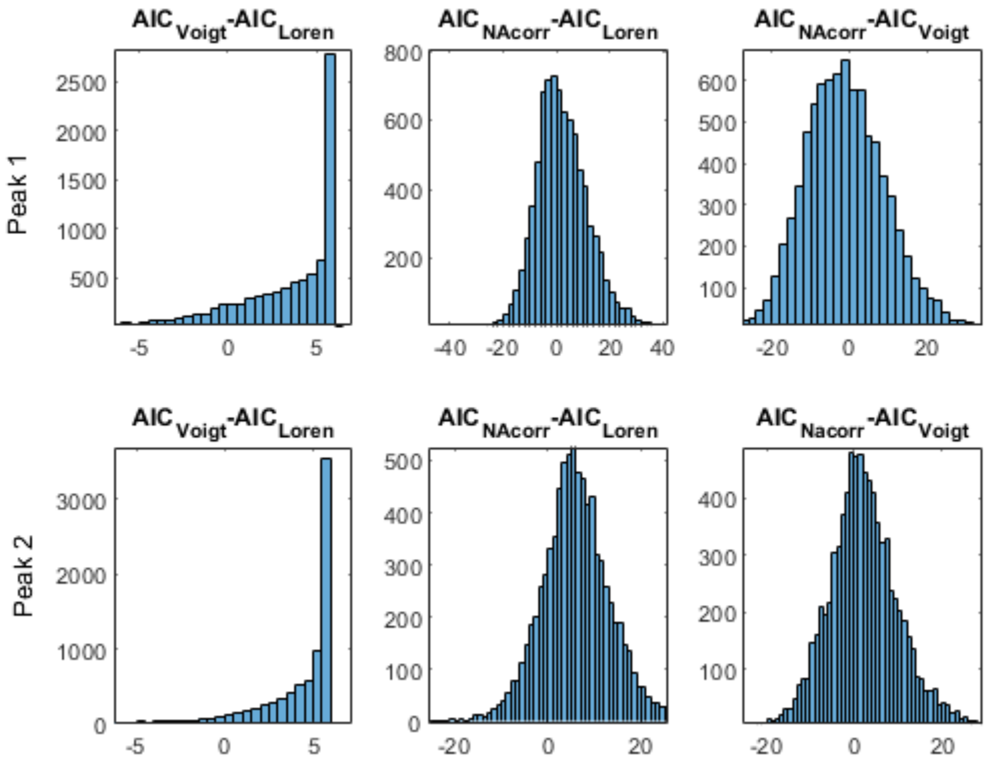

Supplementary Figure 4: Selection of appropriate model for fitting the Brillouin spectra. Histogram of the Akaike Information Criterion (AIC)[61] to compare the appropriateness of different models (Lorentzian, Voigt and NA-Corrected[62]) in fitting the Brillouin spectra. Here, the larger the deviation of the model AIC from the Lorentzian AIC, the poorer is the fit of the model with respect to the Lorentzian model. Data shows that the Lorentzian fit is better than the Voigt's model on most points, while the NA-Corrected model is an equally good fit compared to the Lorentzian model. Data is from Fig 3a - middle panel (incipient antral).

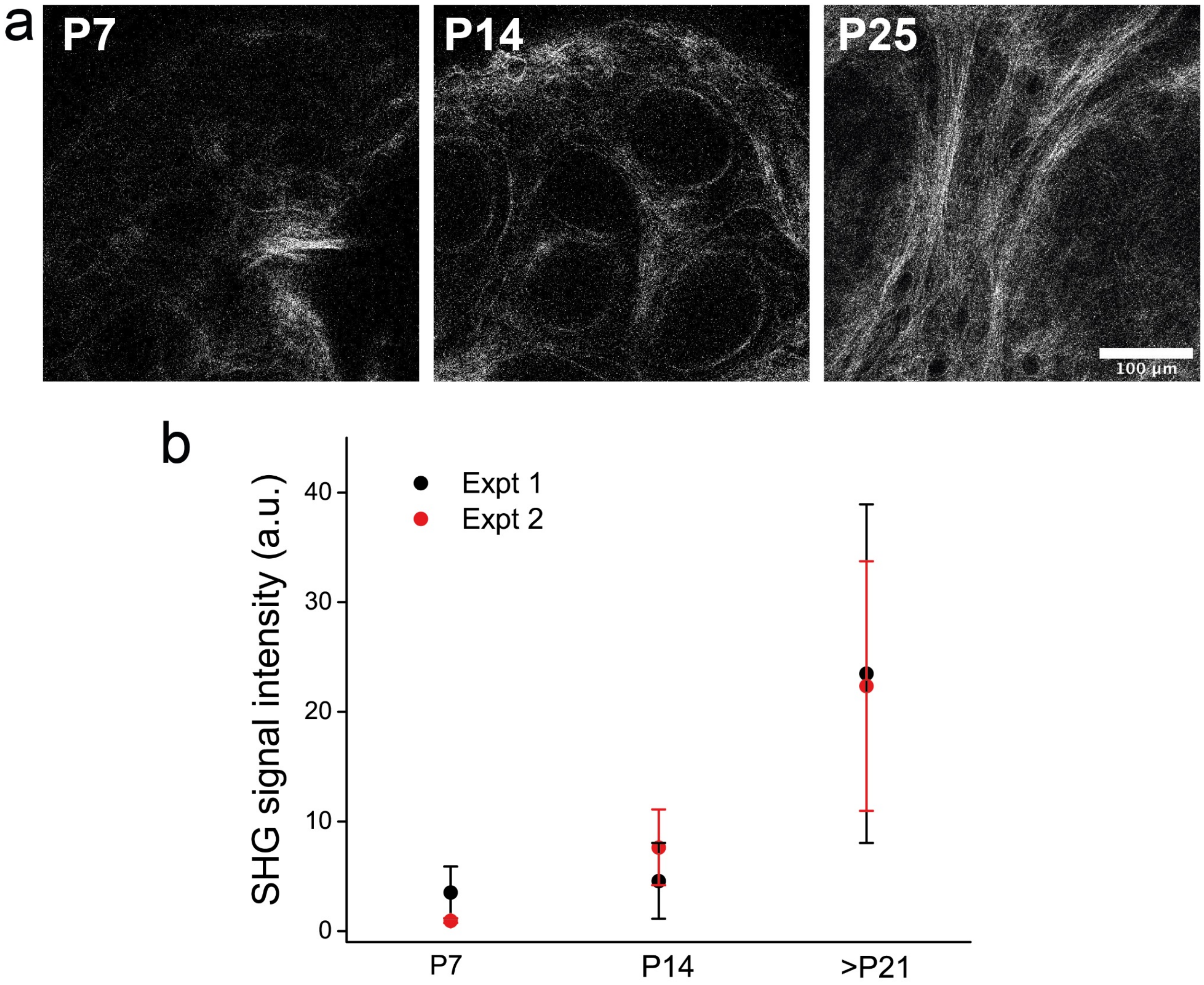

Supplementary Figure 5: Second Harmonic Generation imaging of fibrillar collagen deposition in mouse ovaries of various stages of folliculogenesis. a. Representative images of P7, P14 and P25 mouse ovaries. **b.** Plot of interstitial SGH signal intensity with follicle maturation, indicating that the interstitial collagen network becomes denser and more fibrillar during follicle maturation. PF: Primordial follicle, SF: Secondary follicle, AF: Antral follicle. *N* = 9 from four P7 ovaries, *N* = 17 from four P14 ovaries, and *N* = 23 from four P25 ovaries. Error bars denote S.D.

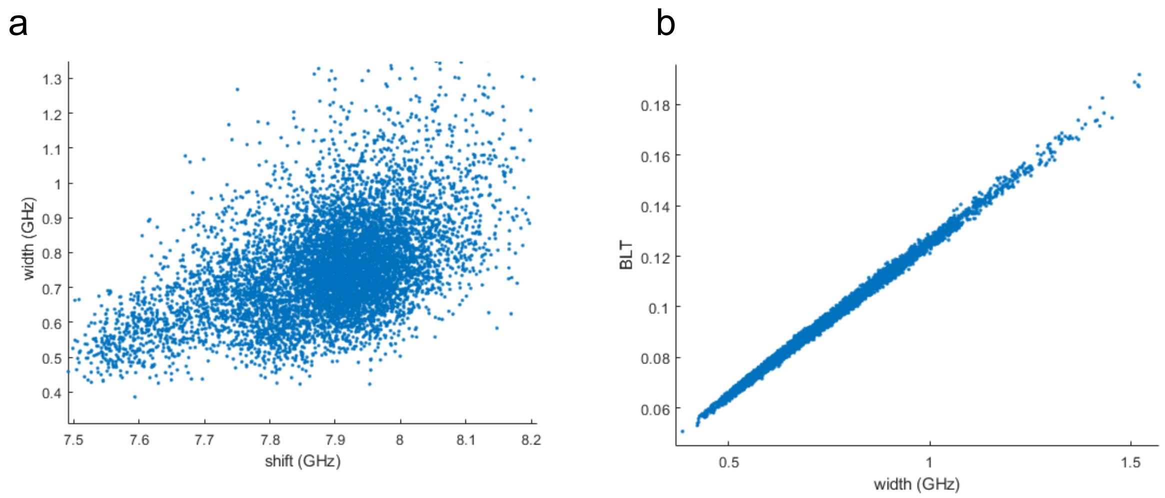

Supplementary Figure 6: Analysis of Brillouin spectra in an ovarian tissue. a. Plot of Brillouin linewidth versus shift in an image, using Lorentzian fit. **b.** Plot of Brillouin Loss Tangent (BLT) versus the linewidth in the same image, showing strong correlation between the two parameters. Each data point corresponds to a pixel on the image. In (a), the data shows poor correlation between the width and shift in the ovarian tissue region (>7.75 GHz in the shift) suggesting heterogeneous broadening, while a better correlation between the shift and width is observed in the lower shift region (<7.75GHz), as expected for the medium and the hydrated part of the tissue[52,53]. Data is from Fig. 3 - middle panel.
