# Supplementary material for: Mechanical mapping of mammalian follicle development using Brillouin microscopy
Source: Commun Biol. 2021 Sep 27;4:1133. doi: 10.1038/s42003-021-02662-5 (PMC8476509; doi:10.1038/s42003-021-02662-5)
Supplement: Supplementary file 2 — Description of Additional Supplementary Files [file 42003_2021_2662_MOESM2_ESM.pdf]

Description of Additional Supplementary Files

File name: Supplementary Data 1.
Description: Raw data for Fig. 3c.

File name: Supplementary Data 2.
Description: Raw data for Fig. 4c.
