# Supplementary material for: Mechanical mapping of mammalian follicle development using Brillouin microscopy
Source: Commun Biol. 2021 Sep 27;4:1133. doi: 10.1038/s42003-021-02662-5 (PMC8476509; doi:10.1038/s42003-021-02662-5)
Supplement: Supplementary file 5 — Reporting Summary [file 42003_2021_2662_MOESM5_ESM.pdf]

nature research

Corresponding author(s): Chii Jou Chan, Robert Prevedel

Reporting Summary

Nature Research wishes to improve the reproducibility of the work that we publish. This form provides structure for consistency and transparency in reporting. For further information on Nature Research policies, see Authors & Referees and the Editorial Policy Checklist.

Statistical parameters

When statistical analyses are reported, confirm that the following items are present in the relevant location (e.g. figure legend, table legend, main text, or Methods section).

n/a	Confirmed	
☐	☒	The exact sample size (*n*) for each experimental group/condition, given as a discrete number and unit of measurement
☐	☒	An indication of whether measurements were taken from distinct samples or whether the same sample was measured repeatedly
☐	☒	The statistical test(s) used AND whether they are one- or two-sided<br>*Only common tests should be described solely by name; describe more complex techniques in the Methods section.*
☒	☐	A description of all covariates tested
☒	☐	A description of any assumptions or corrections, such as tests of normality and adjustment for multiple comparisons
☒	☐	A full description of the statistics including central tendency (e.g. means) or other basic estimates (e.g. regression coefficient) AND variation (e.g. standard deviation) or associated estimates of uncertainty (e.g. confidence intervals)
☐	☒	For null hypothesis testing, the test statistic (e.g. *F*, *t*, *r*) with confidence intervals, effect sizes, degrees of freedom and *P* value noted<br>*Give P values as exact values whenever suitable.*
☒	☐	For Bayesian analysis, information on the choice of priors and Markov chain Monte Carlo settings
☒	☐	For hierarchical and complex designs, identification of the appropriate level for tests and full reporting of outcomes
☒	☐	Estimates of effect sizes (e.g. Cohen's *d*, Pearson's *r*), indicating how they were calculated
☐	☒	Clearly defined error bars<br>*State explicitly what error bars represent (e.g. SD, SE, CI)*

Our web collection on statistics for biologists may be useful.

Software and code

Policy information about availability of computer code

Data collection	Custom written LabView 2015 microscope control software
Data analysis	Custom written Matlab 2018a,b scripts. Code is available from the corresponding authors on request. Statistical analysis was performed using the software Origin (version 8.5.6; OriginLab)

For manuscripts utilizing custom algorithms or software that are central to the research but not yet described in published literature, software must be made available to editors/reviewers upon request. We strongly encourage code deposition in a community repository (e.g. GitHub). See the Nature Research guidelines for submitting code & software for further information.

Data

Policy information about availability of data

All manuscripts must include a data availability statement. This statement should provide the following information, where applicable:

- Accession codes, unique identifiers, or web links for publicly available datasets
- A list of figures that have associated raw data
- A description of any restrictions on data availability

The datasets generated and analysed in the current study are available from the corresponding authors upon reasonable request. Raw data for Fig. 3c and 4c are presented in Supplementary Data 1 and 2, respectively.

Field-specific reporting

Please select the best fit for your research. If you are not sure, read the appropriate sections before making your selection.

☒ Life sciences ☐ Behavioural & social sciences ☐ Ecological, evolutionary & environmental sciences

For a reference copy of the document with all sections, see nature.com/authors/policies/ReportingSummary-flat.pdf

Life sciences study design

All studies must disclose on these points even when the disclosure is negative.

Sample size	No particular statistical method was used to define sample size. Sample size was determined based on previous studies in the field. A minimum of 3 independent experiments were carried out.
Data exclusions	Images with a too low signal to noise ratio of the Brillouin shift and width were excluded. Representative data sets were chosen for the Figures.
Replication	Experiments were reliably reproduced. At least 3 independent experiments were carried out. Experiments were performed on different days with different batches of ovaries.
Randomization	No randomization was applied.
Blinding	Authors were blinded to any group allocation since the mice were chosen randomly for the study.

Reporting for specific materials, systems and methods

Materials & experimental systems

n/a	Involved in the study	
☒	☐	Unique biological materials
☐	☒	Antibodies
☒	☐	Eukaryotic cell lines
☒	☐	Palaeontology
☐	☒	Animals and other organisms
☒	☐	Human research participants

Methods

n/a	Involved in the study	
☒	☐	ChIP-seq
☒	☐	Flow cytometry
☒	☐	MRI-based neuroimaging

Antibodies

Antibodies used	Primary pAb Anti-Collagen IV (Millipore, AB756P) used at 1:100.<br>Secondary antibodies targeting rabbit immunoglobulin-coupled Alexa Fluor 546 (Invitrogen, A10040) was used at 1:200.
Validation	C Anderson, et al. 'Sonic hedgehog-dependent synthesis of laminin alpha1 controls basement membrane assembly in the myotome', Development 136(20):3495-504, 2009

Animals and other organisms

Policy information about studies involving animals; ARRIVE guidelines recommended for reporting animal research

Laboratory animals	Embryonic and postnatal mice ovaries were obtained in the animal facility at the European Molecular Biology Laboratory, with permission from the institutional veterinarian overseeing the operation (ARC number 2020-01-06RP). The animal facilities are operated according to international animal welfare rules (Federation for Laboratory Animal Science Associations guidelines and recommendations).
Wild animals	The study did not involve wild animals.
Field-collected samples	The study did not involve field-collected samples.
